# Hearing Loss and Associated 7-Year Cognitive Outcomes Among Hispanic and Latino Adults

**DOI:** 10.1001/jamaoto.2024.0184

**Published:** 2024-03-21

**Authors:** Ariana M. Stickel, Alonzo Mendoza, Wassim Tarraf, Sayaka Kuwayama, Sonya Kaur, Alejandra Morlett Paredes, Martha L. Daviglus, Fernando D. Testai, Donglin Zeng, Carmen R. Isasi, Rachael R. Baiduc, Elizabeth Dinces, David J. Lee, Hector M. González

**Affiliations:** 1 Department of Psychology, San Diego State University, San Diego, California; 2 Department of Neurosciences, University of California, San Diego, La Jolla; 3 Institute of Gerontology & Department of Healthcare Sciences, Wayne State University, Detroit, Michigan; 4 Department of Neurology, University of Miami Miller School of Medicine, Miami, Florida; 5 Institute for Minority Health Research, College of Medicine, University of Illinois at Chicago, Chicago; 6 Department of Neurology & Rehabilitation, College of Medicine, University of Illinois at Chicago, Chicago; 7 Department of Biostatistics, University of North Carolina, Chapel Hill; 8 Department of Epidemiology & Population Health, Albert Einstein College of Medicine, Bronx, New York; 9 Speech, Language, and Hearing Sciences, University of Colorado Boulder, Boulder; 10 Department of Otorhinolaryngology, Albert Einstein College of Medicine, Bronx, New York; 11 Department of Epidemiology & Public Health, University of Miami, Miami, Florida

## Abstract

**Importance:**

Hearing loss appears to have adverse effects on cognition and increases risk for cognitive impairment. These associations have not been thoroughly investigated in the Hispanic and Latino population, which faces hearing health disparities.

**Objective:**

To examine associations between hearing loss with 7-year cognitive change and mild cognitive impairment (MCI) prevalence among a diverse cohort of Hispanic/Latino adults.

**Design, Setting, and Participants:**

This cohort study used data from a large community health survey of Hispanic Latino adults in 4 major US cities. Eligible participants were aged 50 years or older at their second visit to study field centers. Cognitive data were collected at visit 1 and visit 2, an average of 7 years later. Data were last analyzed between September 2023 and January 2024.

**Exposure:**

Hearing loss at visit 1 was defined as a pure-tone average (500, 1000, 2000, and 4000 Hz) greater than 25 dB hearing loss in the better ear.

**Main outcomes and measures:**

Cognitive data were collected at visit 1 and visit 2, an average of 7 years later and included measures of episodic learning and memory (the Brief-Spanish English Verbal Learning Test Sum of Trials and Delayed Recall), verbal fluency (word fluency—phonemic fluency), executive functioning (Trails Making Test–Trail B), and processing speed (Digit-Symbol Substitution, Trails Making Test–Trail A). MCI at visit 2 was defined using the National Institute on Aging-Alzheimer Association criteria.

**Results:**

A total of 6113 Hispanic Latino adults were included (mean [SD] age, 56.4 [8.1] years; 3919 women [64.1%]). Hearing loss at visit 1 was associated with worse cognitive performance at 7-year follow-up (global cognition: β = −0.11 [95% CI, −0.18 to −0.05]), equivalent to 4.6 years of aging and greater adverse change (slowing) in processing speed (β = −0.12 [95% CI, −0.23 to −0.003]) equivalent to 5.4 years of cognitive change due to aging. There were no associations with MCI.

**Conclusions and relevance:**

The findings of this cohort study suggest that hearing loss decreases cognitive performance and increases rate of adverse change in processing speed. These findings underscore the need to prevent, assess, and treat hearing loss in the Hispanic and Latino community.

## Key Points

**Question:**

Is hearing loss associated with cognitive outcomes (performance, change, and/or mild cognitive impairment) 7 years later among middle-aged and older Hispanic and Latino adults?

**Findings:**

In this cohort study of 6113 Hispanic and Latino adults, hearing loss was associated with worse cognitive performance 7 years later and adverse change (slowing) in processing speed. The latter was equivalent to the expected slowing over more than 5 years of aging.

**Meaning:**

These results suggest that hearing loss may be detrimental to a wide range of cognitive factors among Hispanic and Latino individuals; processing speed may be particularly susceptible to adverse cognitive change in the presence of hearing loss.

## Teaser

This cohort study of Hispanic and Latino adults examines the association of hearing loss with cognitive change and mild cognitive impairment over a 7-year follow-up.

## Introduction

According to the Lancet Commission, hearing loss accounts for more cases of dementia than any other single modifiable risk factor.^1^ Emerging evidence from a study of largely non-Hispanic Black and White individuals suggests that hearing aid use may help lower dementia incidence among those who are at high risk for the neurological disorder (eg, individuals with high cardiovascular disease risk).^2^ However, studies that connect hearing loss to cognitive decline primarily sample non-Hispanic White individuals,^3,4,5^ and there is a dearth of research on Hispanic and Latino adults. Hispanic and Latino adults are projected to have the largest increase in dementia cases by 2060 among any ethnic or racial group in the US^6^ and are vulnerable to hearing loss.^7^ According to data from the Hispanic Community Health Study/Study of Latinos (HCHS/SOL), prevalence of hearing impairment among Hispanic and Latino adults ages 45 years and older ranges from 18% in Mexican women to 41% in Puerto Rican men.^8^

Among Hispanic and Latino adults, hearing loss, including at subclinical levels, is associated with worse performance on cognitive testing.^9,10^ Notably, these associations were not fully accounted for by other factors (eg, depressive symptoms, socioeconomic status).^9,10,11^ Additionally, we previously reported that hearing loss was associated with poorer learning, memory, and processing speed and executive functioning, and these associations were maintained when accounting for cardiovascular health.^11^

Taken together, this provides mounting evidence linking hearing loss to lower cognitive functioning among middle-aged and older Hispanic and Latino adults. However, existing findings are largely based on cross-sectional data and do not disentangle premorbid differences in cognitive abilities from increased risk for cognitive declines and mild cognitive impairment (MCI). To fill this scientific gap, we examined the associations between hearing loss with 7-year cognitive change and MCI risk. We predicted that individuals with hearing loss would have lower cognitive scores, greater adverse cognitive change, and higher prevalence of MCI.

## Methods

### Participants

The HCHS/SOL is a multisite, population-based, prospective cohort study of diverse Hispanic and Latino adults (visit 1 occurring between 2008 and 2011). A total of 16 415 self-identified Hispanic and Latino adults (ages 18-74 years) were sampled from 4 major US metropolitan areas: Bronx, New York; Chicago, Illinois; Miami, Florida; and San Diego, California.^12,13^ Institutional review boards at each site approved this study, and participants provided written informed consent. This study followed the Strengthening the Reporting of Observational Studies in Epidemiology (STROBE) reporting guideline.

The Study of Latinos-Investigation of Neurocognitive Aging (SOL-INCA) is an HCHS/SOL ancillary study that examines neurocognition among a subset of participants who underwent neurocognitive testing at visit 1, completed HCHS/SOL visit 2, and were ages 50 years and older. eFigure 1 in Supplement 1 illustrates how the 6377 participants in SOL-INCA were determined. SOL-INCA (referred to throughout as visit 2) study design and sampling procedures are published elsewhere.^14^ HCHS/SOL generated weights based on complex survey design and sampling procedures to obtain target area representative data of diverse Hispanic and Latino adults.

### Measures

Hearing loss was defined as pure tone average (PTA) (including 500, 1000, 2000, and 4000 Hz) above 25 dB hearing level (dB HL) in the better ear.^7,11^ For outcomes, we used a standardized cognitive test battery across sites that was administered by bilingual (Spanish-English), bicultural staff. Cognitive tests were selected based on use in other population-based samples^15^ and/or appropriateness for the Hispanic and Latino population.^14^ Cognitive measures at visit 2 included learning and memory (Brief-Spanish English Verbal Learning Test [B-SEVLT] Sum of Trials and Delayed Recall), processing speed (Digit-Symbol Substitution [DSS] and Trails Making Test–Trail A), executive functioning (Trails Making Test–Trail B), and phonemic word fluency.^14,16,17,18,19^ Higher scores on Trails A and B reflect slower performance. All measures were *z* scored. A global cognition score was generated by taking the average of *z*-scored individual tests (excluding Trails A and B).

Cognitive change scores were calculated in line with simple standardized regression-based techniques for assessing change^20^ and were done for measures that were repeated from HCHS/SOL, henceforth visit 1 (ie, B-SEVLT learning and memory, DSS, word fluency). Change in global cognition was also calculated from the repeated tests. Briefly, we used weighted linear regressions to estimate cognitive performance at visit 2 as a function of performance at visit 1, adjusting for time elapsed (in days) between cognitive assessments.^20,21^ Then test-specific standardized measures of change were calculated using the formula (test 2 − test 2 estimate)/RMSE, where test 2 was the respondent cognitive score at visit 2 and RMSE is the root mean squared error.^22^ The generated *z* scores reflect global and test-specific change in the target population of SOL-INCA. Scores below zero denote cognitive decline or performance lower than expectation at visit 2 in *z* score units, while scores of zero or higher indicate no change or improvement in cognitive function.

Because the B-SEVLT is a verbally presented list of words that may affect performance for individuals with hearing loss, we also measured recall as total recall divided by the highest score from the 3 learning trials (ie, B-SEVLT-Recall/maximum score of 3 trials) per participant. This recall score (also *z*-scored) was tested as an individual outcome in all performance and cognitive change models, but was not included in the development of the global cognition composite. MCI (yes, no) was operationalized using National Institute on Aging-Alzheimer Association criteria as defined by the SOL-INCA study, which have been previously published.^23,24,25^

Covariates included sex (female, male), age in years, education (less than high school, high school or equivalent, more than high school), self-reported Hispanic or Latino heritage (Dominican, Central American, Cuban, Mexican, Puerto Rican, South American, other), field center (Bronx, Chicago, Miami, San Diego), income (less than or equal to $20 000, $20 001 to $50 000, greater than $50 000, not reported), marital status (single; married or living with partner; separated, divorced, or widowed), depressive symptoms (using the Center for Epidemiological Studies-Depression 10-item questionnaire [CES-D10]), cardiovascular disease risk score (Framingham Cardiovascular Disease 10-year risk), total cholesterol (mg/dL; to convert to millimoles per liter, multiply by 0.0259), triglycerides (mg/dL; to convert to millimoles per liter, multiply by 0.0113), and fasting glucose (mg/dL; to convert to millimoles per liter, multiply by 0.0555). All covariates were measured at visit 1.

### Analytic Samples

We excluded 264 individuals who had missing exposures or covariate data. Cognitive performance at visit 2 and cognitive change analytic sample comprised 6113 individuals. The MCI prevalence analytic sample was 6031 individuals after further excluding 82 individuals with suspected severe cognitive impairment.^23^

### Statistical Analysis

All analyses accounted for the survey design elements (probability weights, clustering, and stratification) at SOL-INCA to generate correct variance estimates as well as weighted statistics (eg, means and β coefficients) reflecting the target population of SOL-INCA. Detailed discussion of the implications of complex sample design corrections are published elsewhere.^26^ First, we reported descriptive statistics by hearing loss status for the 2 different analytic samples addressed above (cognition and MCI outcomes). We reported weighted percentages and 95% CIs for categorical variables and means and their corresponding confidence intervals for continuous measures. Second, we used survey generalized linear regressions for the continuous outcomes (ie, cognitive scores at visit 2 and cognitive change) and survey logistic regressions for MCI prevalence outcomes controlling for covariates. We estimated 95% CIs of regression coefficients for the models with continuous outcomes (ie, cognitive scores at visit 2 and cognitive change) and odds ratios for MCI prevalence. To highlight potential substantive and clinical significance of our findings, we anchored the estimated coefficients of the hearing exposures relative to our age covariable independently in each model by dividing the coefficient for the hearing variable by the age estimate (when both variables are associated with the outcomes). This translated the hearing estimates into age-specific units (eg, hearing loss is equivalent to the calculated number years of cognitive aging).

We conducted 4 sets of sensitivity models to expand on the operational definition of hearing loss. First, we replaced hearing loss with continuous PTA. Second, we replicated the main analyses and the first set of sensitivity models while excluding individuals who reported ever using a hearing aid (82 individuals [61 identified as having hearing loss and 21 identified as not experiencing hearing loss]).

Third, we operationalized hearing loss clinical severity by creating 3 groups reflecting normal (PTA of 20 dB HL or less), mild (PTA between less than 20 and 40 dB HL), and moderate plus (greater than 40 dB HL; moderate, severe, and profound categories were collapsed into 1 group due to small cells sizes).^27^ Fourth, we operationalized hearing loss reconciling the clinical hearing diagnostic criteria and our original definition for hearing loss (defined as PTA above 25 dB HL). The resulting groups consisted of normal (PTA of 20 dB or below), mild but not impaired (PTA between less than 20 and 25 dB HL), and hearing loss (PTA greater than 25 dB HL). Data were last analyzed between September 2023 and January 2024.

## Results

A total of 6113 adults were included in the analysis (mean [SD] age, 56.4 [8.1] years; 3919 women [64.1%]). Hearing loss (836 of 6113 participants [14%]) was associated with older age (mean age: with hearing loss, 61.7 [95% CI, 60.8-62.5] years vs without hearing loss, 55.4 [95% CI, 55.0-55.7] years), male sex (60.4% [95% CI, 55.8%-65.1%] vs 39.6% [95% CI, 34.9%-44.2%]), income less than or equal to $20 000 (56.0% [95% CI, 50.4%-61.3%] vs $50 001 or more, 4.3% [95% CI, 2.7%-6.0%]), higher mean Framingham risk score (24.3 [95% CI, 22.5-26.2] vs 14.4 [95% CI, 13.8-15.0]), and higher mean glucose (116.9 [95% CI, 112.9-120.9] vs 108.3 [95% CI, 106.8-109.8]) (Table 1). Patterns were similar in the MCI outcome analytic sample (eTable 1 in Supplement 1). The mean (SD) time between cognitive testing visits was 6.9 (1.2) years.

**Table 1.** Descriptive Statistics for the Study of Latinos-Investigation of Neurocognitive Aging Overall Sample^a^

Variable	Hearing loss, % (95% CI)		
	No (n = 5277)	Yes (n = 836)	Total (N = 6113)
**Categorical variables**			
Sex			
Female	57.1 (55.3-58.9)	39.6 (34.9-44.2)	54.2 (52.4-55.9)
Male	42.9 (41.1-44.8)	60.4 (55.8-65.1)	45.9 (44.1-47.6)
Education			
Less than high school	36.8 (34.5-39.1)	45.9 (41.4-50.5)	38.3 (36.2-40.4)
High school or equivalent	21.55 (20.0-23.1)	19.2 (15.2-23.3)	21.2 (19.7-22.7)
More than high school	41.7 (39.5-43.29)	34.8 (30.0-39.6)	40.5 (38.5-42.5)
Income, $			
≤20 000	45.1 (42.6-47.6)	56.0 (50.4-61.6)	46.9 (44.3-49.5)
20 001-50 000	35.5 (33.6-37.5)	29.1 (24.3-33.9)	34.5 (32.5-36.4)
≥50 001	10.9 (9.0-12.7)	4.3 (2.7-6.0)	9.8 (8.2-11.3)
Not reported	8.5 (7.2-9.9)	10.6 (7.8-13.3)	8.9 (7.6-10.1)
Marital status			
Single	16.9 (15.3-18.5)	15.5 (11.9-19.1)	16.7 (15.2-18.1)
Married or living with partner	54.8 (52.3-57.3)	53.9 (48.8-58.9)	54.6 (52.2-57.0)
Separated, divorced, or widowed	28.3 (26.2-30.4)	30.6 (26.3-34.9)	28.7 (26.8-30.6)
Field center			
Bronx, New York	26.9 (24.0-29.9)	24.2 (19.1-29.4)	26.5 (23.5-29.4)
Chicago, Illinois	12.7 (11.0-14.4)	11.4 (8.7-14.2)	12.5 (10.8-14.2)
Miami, Florida	35.2 (30.8-39.7)	43.1 (35.9-50.3)	36.5 (32.0-41.1)
San Diego, California	25.2 (21.8-28.6)	21.3 (16.7-25.8)	24.5 (21.2-27.8)
Heritage			
Dominican	9.5 (7.9-11.1)	7.2 (5.0-9.3)	9.1 (7.6-10.6)
Central American	7.3 (6.1-8.6)	7.3 (5.0-9.7)	7.3 (6.2-8.5)
Cuban	24.8 (21.1-28.5)	32.1 (25.7-38.5)	26.0 (22.3-29.8)
Mexican	34.8 (31.4-38.2)	26.9 (21.8-32.1)	33.5 (30.1-36.9)
Puerto Rican	14.5 (12.9-16.2)	18.6 (15.1-22.1)	15.2 (13.6-16.8)
South American	5.3 (4.5-6.1)	4.7 (2.7-6.7)	5.2 (4.5-6.0)
Other	3.7 (2.8-4.7)	3.2 (1.4-5.0)	3.6 (2.8-4.5)
Continuous variables			
Age, mean (95% CI), y	55.4 (55.0-55.7)	61.7 (60.8-62.5)	56.4 (56.0-56.8)
Depressive symptoms from CESD, mean (95% CI)	7.3 (7.1-7.6)	7.6 (6.9-8.3)	7.4 (7.1-7.6)
Framingham CVD–10 y risk, mean (95% CI)	14.4 (13.8-15.0)	24.3 (22.5-26.2)	16.1 (15.4-16.8)
Total cholesterol, mean (95% CI), mg/dL	209.9 (208.1-211.6)	204.4 (198.8-209.9)	209.3 (207.5-211.0)
Triglycerides, mean (95% CI), mg/dL	147.9 (144.2-151.6)	158.5 (148.3-168.8)	151.2 (146.6-155.8)
Glucose (fasting), mean (95% CI), mg/dL	108.3 (106.8-109.8)	116.9 (112.9-120.9)	109.7 (108.2-111.2)

Abbreviations: CESD, Center for Epidemiology Studies Depression Scale; CVD, cardiovascular disease.

SI conversion factors: To convert cholesterol to millimoles per liter, multiply by 0.0259; triglycerides to millimoles per liter, multiply by 0.0113; fasting glucose to millimoles per liter, multiply by 0.0555.

^a^ Sample size is unweighted, and all other reported values are weighted to represent the target Hispanic and Latino population. Variables are measured at visit 1.

### Primary Analyses

Hearing loss was associated with lower global cognition at visit 2 (β = −0.11 [95% CI, −0.18 to −0.05]; equivalent to 4.6 years of aging) and lower cognitive domain-specific scores, including learning, memory, and word fluency (Table 2; eFigure 2 in Supplement 1). In models that operationalized memory as a proportion recalled from learned items (B-SEVLT-Recall/maximum score of 3 trials), hearing loss was not associated with memory. Hearing loss was only associated with adverse change in processing speed (DSS: β = −0.12 [95% CI, −0.23 to −0.003]; equivalent to 5.4 years of cognitive decline due to aging) (Table 2; eFigure 3 in Supplement 1). Hearing loss status was not associated with MCI prevalence (odds ratio, 0.91 [95% CI, 0.65-1.26]).

**Table 2.** Associations Between Hearing Loss at Visit 1 With Cognitive Performance at Visit 2 and Cognitive Change^a^

Outcome	β (95% CI)							
	B-SEVLT-Sum	B-SEVLT-Recall	Word fluency	Digit and symbol substitution	Trails A	Trails B	Global cognition	B-SEVLT-Recall/maximum of 3 trials
**Cognitive outcomes** ^b^								
Hearing loss								
No	[Reference]	[Reference]	[Reference]	[Reference]	[Reference]	[Reference]	[Reference]	[Reference]
Yes	−0.14 (−0.24 to −0.04)	−0.12 (−0.22 to −0.02)	−0.12 (−0.23 to −0.01)	−0.07 (−0.15 to 0.02)	0.07 (−0.03 to 0.17)	0.02 (−0.08 to 0.12)	−0.11 (−0.18 to −0.05)	0.02 (−0.07 to 0.11)
Unweighted, No.	6091	6080	6072	6031	6036	5816	6091	6086
**Change from visit 1 to visit 2**								
Hearing loss								
No	[Reference]	[Reference]	[Reference]	[Reference]	NA	NA	[Reference]	[Reference]
Yes	−0.11 (−0.22 to 0.01)	−0.10 (−0.21 to 0.01)	−0.06 (−0.18 to 0.06)	−0.12 (−0.23 to −0.00)	NA	NA	−0.11 (−0.22 to 0.01)	−0.10 (−0.21 to 0.02)
Unweighted No.	6061	6054	5976	5910	NA	NA	6078	6053

Abbreviations: B-SEVLT, Brief- Spanish English Verbal Learning Test; NA, not applicable.

^a^ Models adjust for sex, age, education, heritage, center, income, marital status, depressive symptoms, Framingham cardiovascular disease−10 y risk, total cholesterol, triglycerides, and fasting glucose.

^b^ Cognitive outcomes are *z* scored.

### Sensitivity Analyses

We found largely similar patterns as observed with categorical hearing status with some evidence of additional links to the cognitive outcomes (performance at visit 2, cognitive change). Specifically, higher PTA (worse hearing sensitivity) was additionally associated with slower performance on both processing speed measures (DSS: β = −0.004 [95% CI, −0.007 to −0.001]; Trail A: β = 0.005 [95% CI, 0.002 to 0.008]; equivalent of 1 and 1.7 years of cognitive aging, respectively) (eTables 2 and 3 in Supplement 1). Additionally, higher (ie, worse) hearing sensitivity was associated with an adverse change in learning (β = −0.004 [95% CI, −0.008 to −0.0004]; equivalent to 2 years of cognitive decline due to aging).

In the second sensitivity analysis, which excluded individuals with history of hearing aid use, descriptive statistics highly resembled the full sample (eTable 4 in Supplement 1). Estimates remained largely unchanged when compared with the results from the full sample (eTables 5-8 in Supplement 1). We found additional evidence for an association between hearing loss and adverse change in global cognition (β = −0.12 [95% CI, −0.23 to −0.0003]; equivalent of 5 years of aging). However, the association between hearing loss and adverse change in processing speed (DSS) in the full sample analysis was lost when excluding individuals with history of hearing aid use.

In the analysis of hearing loss grouped by normal, mild, and moderate levels, we observed similar patterns reflective of the binary hearing loss status with nominal evidence of dose effects moving from normal to mild to moderate plus with respect to the B-SEVLT Sum and Recall and global cognition, but not for word fluency (eTable 9 in Supplement 1). We found no associations with cognitive change or MCI (eTable 10 in Supplement 1). The threshold for hearing loss (ie, greater than 25 dB HL) was more sensitive to detecting cognitive underperformance on word fluency and change in processing speed (DSS) compared with the threshold for mild loss (ie, greater than 20 dB HL), which is consistent with the primary results (eTables 11 and 12 in Supplement 1).

## Discussion

To summarize the results, we found evidence that hearing loss was associated with worse cognitive performance, and to a lesser extent greater change in cognition, at 7-year follow-up among a diverse group of over 6000 Hispanic and Latino adults. We extended previous HCHS/SOL cross-sectional findings that linked hearing loss to worse concurrent cognitive performance^10^ now to worse performance in learning, memory, and overall global cognition an average 7 years later. Several studies have also related hearing loss to worse cognitive performance with executive functioning, learning, and memory differences most often documented.^11,28,29,30,31,32^ Curiously, we only observed connections between hearing and later processing speed and/or executive functions when taking into account a continuous measure of hearing threshold, suggesting a linear, insidious relationship between hearing loss and this cognitive domain.^9^ Regarding verbal memory performance at visit 2, we observed mixed evidence with hearing loss when taking into account words initially learned. This suggests that, to a certain extent, the present verbal memory results are affected by the participant’s ability to hear and should be carefully considered in research and clinical work.

Hearing-loss–related changes in processing speed were equivalent to more than 5 years of aging. Uchida and colleagues^33^ similarly observed accelerated rates of decline on the DSS among those with hearing loss. Although hearing loss has been related to greater adverse changes in memory,^34^ the present study and others have found that this association is not robust.^30^ Our previous work^11^ suggested that the combination of hyperglycemia and hearing loss was particularly detrimental to learning and memory. Taken together, hearing loss and cardiovascular disease risk factors may have overlapping and potentially synergistic impacts on rate of change in memory.

Hearing loss has been linked to changes to the brain, which may have implications for changes in cognition across various domains. For example, hearing loss has been associated with smaller brain volumes and lower white matter microstructural integrity,^35,36,37^ and this could slow cognitive processing.^38^ Additionally, hearing loss has been associated with tau pathology, an early indicator of Alzheimer disease.^37^ Other hearing loss–related changes to the brain may include dysfunctional neurotransmission due to decreased levels of GABA receptors, cortical thinning, and reduced gray matter volume, particularly in primary auditory cortex within the temporal lobe.^39,40^

The role of ototoxic exposures in the observed hearing-loss–related cognitive differences is unknown. Mouse models with noise and/or ototoxin-related damage to hair cells demonstrate decreased neurogenesis in the hippocampus and deficits in spatial memory.^41,42^ In humans, certain ototoxins (eg, lead)^43^ are associated with structural brain differences and cognitive deficits in addition to increasing risk for Alzheimer disease.^44,45,46,47^ Importantly, Hispanic and Latino adults, especially immigrants, tend to be overrepresented in occupations and environments that can lead to hearing loss (eg, via exposures to ototoxic chemicals and/or loud noises).^48,49,50^ Exposure to and treatment for such occupational hazards are influenced by a variety of employer-driven factors, including availability of effective safety training in both English and Spanish, availability of safety equipment, enforcement of Occupational Safety and Health Administration regulations, and employee access to health care benefits to treat ototoxic exposure should it occur.^51,52^ Therefore, future research should investigate whether more systemwide protections for disenfranchised employees would prevent hearing loss and related long-term cognitive disadvantages.

In contrast with the majority of existing research,^53,54,55,56^ we did not detect significant hearing loss–related differences in MCI risk. Both objective and subjective measures of hearing loss have been associated with higher rates of cognitive impairment (MCI and dementia) and across individuals of varying nationalities and ethnicities, although there is significant underrepresentation of Hispanic and Latino individuals.^53,54,55,56,57,58^ Indeed, in a 2022 meta-analysis,^59^ hearing loss was associated with increased risk for MCI. The differing findings in this study may be related to our definition of MCI, which tends to be more conservative than many studies that base their operationalization on a brief cognitive screener rather than robust normative data on a variety of cognitive tests. We also considered several confounders from sociodemographics to cardiovascular disease risk factors. Controlling for similar covariates attenuated the association between baseline hearing loss and prevalent, but not incident, cognitive impairment (MCI or dementia) among middle-aged and older Singaporeans.^60^ Certain cardiovascular disease risk factors (eg, diabetes) are more prevalent among Hispanic and Latino adults compared with non-Hispanic Asian and White adults, and these are risk factors for MCI.^61,62,63,64^ Additionally, compared with many existing studies^53,54,55^ our cohort is relatively young, which lowers the likelihood of both hearing loss and MCI.^23,65^ Of note, among a subsample of 40- to 64-year-olds, only profound hearing loss (hearing threshold of 90 dB or greater in both ears) was associated with increased risk of dementia when controlling for sociodemographics and cardiovascular disease risk factors.^66^ In our own study, moderate to profound hearing loss was not associated with MCI, although we may lack precision to detect such outcomes given the small number of participants in this hearing loss range.

In the present sample, excluding individuals who reported ever using hearing aids did not change the overall pattern of results. This is likely due to the small sample of individuals reporting any history of hearing aid use at visit 1 (82 individuals) and lack of information on incident hearing aid use, duration of hearing aid use adherence, and hearing aid type. Intervention studies are needed to evaluate if hearing aid use may have downstream benefits to cognitive aging among Hispanic and Latino individuals as has been observed in other studies, particularly among individuals at risk for cognitive decline.^2,67,68,69^

### Limitations

Our study had several limitations. First, incident hearing loss and/or hearing aid use were not tracked. In an Australian sample, 5-year incident hearing loss was 17.9% with a 3-fold increase of risk each decade after 60 years of age.^65^ Second, we did not account for type of hearing loss, which may partially explain our lack of significant differences in cognitive change and MCI prevalence. For example, individuals with noise-induced and mixed hearing loss may be more susceptible to cognitive impairment than individuals with other forms of hearing loss (eg, conductive, sensorineural, presbycusis).^70^ Third, as noted above, data on hearing aid adherence or incident hearing aid use were not collected. Hearing aid use, compared with nonuse, has been associated with better preservation of cognitive abilities among individuals with hearing loss.^68,69^ Fourth, we took a conservative approach with our models, including some potentially overlapping cardiovascular disease risk factors. Fifth, despite using several cognitive tests that have been performed in other studies of hearing loss and cognition,^33,34^ the appropriateness of these measures for individuals with hearing loss may be limited.^71^ To partially address this, we administered cognitive tests to participants in a quiet space and encouraged them to bring and use any sensory aids (eg, hearing aids, glasses). Finally, we did not account for comorbid sensory impairments, which have been associated with additional increased risk of cognitive decline and dementia compared with individuals without dual sensory impairments.^72^

## Conclusion

Among Hispanic and Latino adults, hearing loss was associated with worse cognitive performance 7 years later and greater change (slowing) in processing speed. From a public health standpoint, hearing health and effectively treating hearing loss may prevent long-term cognitive difficulties for Hispanic and Latino adults who have both high hearing loss and dementia disease burden.^4^

## Notes

Supplement 1. **eTable 1.** Descriptive Statistics: Study of Latinos-Investigation of Neurocognitive Aging Mild Cognitive Impairment Subpopulation **eTable 2.** Associations Between Hearing Sensitivity (Pure Tone Average) at Visit 1 With Cognitive Performance at Visit 2, on Average 7 Years Later, and Cognitive Change **eTable 3.** Associations Between Hearing Sensitivity (Pure Tone Average) at Visit 1 With Mild Cognitive Impairment Prevalence at Visit 2 **eTable 4.** Descriptive Statistics for the Study of Latinos–Investigation of Neurocognitive Aging Excluding Hearing Aid Subsample **eTable 5.** Associations Between Hearing Loss at Visit 1 With Cognitive Performance at Visit 2, on Average 7 Years Later, and Cognitive Change After Exclusion of Hearing Aid Subsample **eTable 6.** Associations Between Hearing Loss at Visit 1 With Mild Cognitive Impairment Prevalence at Visit 2 After Exclusion of Hearing Aid Subsample **eTable 7.** Associations Between Hearing Operationalized Continuously and Cognitive Performance at INCA and Cognitive Change, After Exclusion of Hearing Aid Subsample **eTable 8.** Associations Between Hearing Operationalized Continuously and MCI, After Exclusion of Hearing Aid Subsample **eTable 9.** Associations Between 3 Groups Hearing Impairment at Visit 1 With Cognitive Performance at Visit 2, on Average 7 Years Later, and Cognitive Change **eTable 10.** Associations Between 3 Groups Hearing Impairment at Visit 1 With Mild Cognitive Impairment Prevalence at Visit 2 **eTable 11.** Associations Between 3 Group Hearing Impairment at Visit 1 With Cognitive Performance at Visit 2, on Average 7 Years Later, and Cognitive Change **eTable 12.** Associations Between 3 Group Hearing Impairment at Visit 1 With Mild Cognitive Impairment Prevalence at Visit 2 **eFigure 1.** Analytic Sample Diagram **eFigure 2.** Box Plots of Cognitive Performance Scores at Visit 2 in the Primary Analysis **eFigure 3.** Box Plots of Cognitive Change Scores in the Primary Analysis

Supplement 2.
Data Sharing Statement
