# Supplementary material for: Hearing Loss and Associated 7-Year Cognitive Outcomes Among Hispanic and Latino Adults
Source: JAMA Otolaryngol Head Neck Surg. 2024 Mar 21;150(5):385–92. doi: 10.1001/jamaoto.2024.0184 (PMC10958383; doi:10.1001/jamaoto.2024.0184)
Supplement: Supplement 1. — eTable 1. Descriptive Statistics: Study of Latinos-Investigation of Neurocognitive Aging Mild Cognitive Impairment Subpopulation eTable 2. Associations Between Hearing Sensitivity (Pure Tone Average) at Visit 1 With Cognitive Performance at Visit 2, on Average 7 Years Later, and Cognitive Change eTable 3. Associations Between Hearing Sensitivity (Pure Tone Average) at Visit 1 With Mild Cognitive Impairment Prevalence at Visit 2 eTable 4. Descriptive Statistics for the Study of Latinos–Investigation of Neurocognitive Aging Excluding Hearing Aid Subsample eTable 5. Associations Between Hearing Loss at Visit 1 With Cognitive Performance at Visit 2, on Average 7 Years Later, and Cognitive Change After Exclusion of Hearing Aid Subsample eTable 6. Associations Between Hearing Loss at Visit 1 With Mild Cognitive Impairment Prevalence at Visit 2 After Exclusion of Hearing Aid Subsample eTable 7. Associations Between Hearing Operationalized Continuously and Cognitive Performance at INCA and Cognitive Change, After Exclusion of Hearing Aid Subsample eTable 8. Associations Between Hearing Operationalized Continuously and MCI, After Exclusion of Hearing Aid Subsample eTable 9. Associations Between 3 Groups Hearing Impairment at Visit 1 With Cognitive Performance at Visit 2, on Average 7 Years Later, and Cognitive Change eTable 10. Associations Between 3 Groups Hearing Impairment at Visit 1 With Mild Cognitive Impairment Prevalence at Visit 2 eTable 11. Associations Between 3 Group Hearing Impairment at Visit 1 With Cognitive Performance at Visit 2, on Average 7 Years Later, and Cognitive Change eTable 12. Associations Between 3 Group Hearing Impairment at Visit 1 With Mild Cognitive Impairment Prevalence at Visit 2 eFigure 1. Analytic Sample Diagram eFigure 2. Box Plots of Cognitive Performance Scores at Visit 2 in the Primary Analysis eFigure 3. Box Plots of Cognitive Change Scores in the Primary Analysis [file jamaotolaryngolheadnecksurg-e240184-s001.pdf]

Supplemental Online Content

Stickel AM, Mendoza A, Tarraf W, et al. Relationships between hearing loss and 7-year cognitive outcomes among Hispanic/Latino adults. *JAMA Otolaryngol Head Neck Surg.* Published online March 21, 2024. doi:10.1001/jamaoto.2024.0184

eTable 1. Descriptive Statistics: Study of Latino Adults-Investigation of Neurocognitive Aging Mild Cognitive Impairment Subpopulation

eTable 2. Associations Between Hearing Sensitivity (Pure Tone Average) at Visit 1 With Cognitive Performance at Visit 2, on Average 7 Years Later, and Cognitive Change

eTable 3. Associations Between Hearing Sensitivity (Pure Tone Average) at Visit 1 With Mild Cognitive Impairment Prevalence at Visit 2

eTable 4. Descriptive Statistics for the Study of Latino Adults–Investigation of Neurocognitive Aging Excluding Hearing Aid Subsample

eTable 5. Associations Between Hearing Loss at Visit 1 With Cognitive Performance at Visit 2, on Average 7 Years Later, and Cognitive Change After Exclusion of Hearing Aid Subsample

eTable 6. Associations Between Hearing Loss at Visit 1 With Mild Cognitive Impairment Prevalence at Visit 2 After Exclusion of Hearing Aid Subsample

eTable 7. Associations Between Hearing Operationalized Continuously and Cognitive Performance at INCA and Cognitive Change, After Exclusion of Hearing Aid Subsample

eTable 8. Associations Between Hearing Operationalized Continuously and MCI, After Exclusion of Hearing Aid Subsample

eTable 9. Associations Between 3 Groups Hearing Impairment at Visit 1 With Cognitive Performance at Visit 2, on Average 7 Years Later, and Cognitive Change

eTable 10. Associations Between 3 Groups Hearing Impairment at Visit 1 With Mild Cognitive Impairment Prevalence at Visit 2

eTable 11. Associations Between 3 Group Hearing Impairment at Visit 1 With Cognitive Performance at Visit 2, on Average 7 Years Later, and Cognitive Change

eTable 12. Associations Between 3 Group Hearing Impairment at Visit 1 With Mild Cognitive Impairment Prevalence at Visit 2

eFigure 1. Analytic Sample Diagram

© 2024 Stickel AM et al. *JAMA Network Open.*

eFigure 2. Box Plots of Cognitive Performance Scores at Visit 2 in the Primary Analysis
eFigure 3. Box Plots of Cognitive Change Scores in the Primary Analysis

This supplemental material has been provided by the authors to give readers additional information about their work.

© 2024 Stickel AM et al. *JAMA Network Open.*

SUPPLEMENTAL TABLES

eTable 1. Descriptive statistics: Study of Latinos– Investigation of Neurocognitive Aging mild cognitive impairment subpopulation

	Hearing loss		
	**No (N=5,221)**	**Yes (N=810)**	**Total (N=6,031)**
**Categorical Variables**	**% [CI 95]**	**% [CI 95]**	**% [CI 95]**
**Sex**			
Female	56.94 [55.14;58.74]	40.21 [35.42;45.00]	54.23 [52.52;55.95]
Male	43.06 [41.26;44.86]	59.79 [55.00;64.58]	45.77 [44.05;47.48]
**Education**			
Less than high school	36.56 [34.30;38.82]	45.36 [40.50;50.22]	37.98 [35.87;40.10]
High school or equivalent	21.57 [20.00;23.15]	19.16 [15.14;23.19]	21.18 [19.68;22.68]
More than high school	41.87 [39.67;44.06]	35.48 [30.28;40.67]	40.83 [38.82;42.84]
**Income**			
≤$20000	44.92 [42.44;47.41]	55.27 [49.74;60.80]	46.60 [44.04;49.16]
$20001-$50000	35.79 [33.83;37.75]	29.97 [25.15;34.79]	34.85 [32.93;36.76]
≥$50001	10.97 [9.14;12.79]	4.52 [2.80;6.24]	9.92 [8.32;11.52]
Not Reported	8.32 [6.96;9.69]	10.24 [7.60;12.89]	8.63 [7.38;9.89]
**Marital Status**			
Single	16.99 [15.39;18.59]	15.38 [11.61;19.15]	16.73 [15.22;18.24]
Married/ Living with partner	54.84 [52.29;57.40]	54.23 [49.16;59.29]	54.74 [52.34;57.14]
Separated/ Divorced/ Widowed	28.17 [26.06;30.28]	30.40 [25.84;34.95]	28.53 [26.56;30.50]
**Field Center**			
Bronx	26.91 [23.97;29.85]	24.18 [18.97;29.38]	26.47 [23.53;29.42]
Chicago	12.78 [11.06;14.51]	11.84 [9.03;14.66]	12.63 [10.92;14.34]
Miami	35.04 [30.58;39.51]	42.78 [35.68;49.88]	36.29 [31.73;40.86]
San Diego	25.26 [21.84;28.68]	21.20 [16.64;25.77]	24.60 [21.26;27.95]
**Heritage**			
Dominican	9.41 [7.92;10.90]	6.89 [4.81;8.98]	9.01 [7.60;10.41]
Central American	7.37 [6.11;8.63]	7.67 [5.23;10.11]	7.42 [6.27;8.58]
Cuban	24.61 [20.87;28.35]	31.30 [24.94;37.65]	25.69 [21.91;29.48]
Mexican	34.99 [31.58;38.41]	27.12 [21.91;32.32]	33.72 [30.34;37.09]
Puerto-Rican	14.56 [12.92;16.21]	18.78 [15.15;22.42]	15.25 [13.61;16.89]
South American	5.35 [4.54;6.16]	4.92 [2.87;6.97]	5.28 [4.50;6.06]
Other	3.70 [2.72;4.67]	3.31 [1.44;5.19]	3.64 [2.77;4.50]
**Continuous Variables**	**Mean [CI 95]**	**Mean [CI 95]**	**Mean [CI 95]**
**Age in years**	55.27 [54.89;55.65]	61.50 [60.63;62.37]	56.27 [55.90;56.64]
**Depressive symptoms from CESD**	7.26 [7.01;7.50]	7.48 [6.79;8.18]	7.32 [7.08;7.56]
**Framingham CVD - 10yr risk**	14.18 [13.60;14.75]	24.01 [22.18;25.84]	15.81 [15.14;16.48]
**Total cholesterol** mg/dL	209.99 [208.27;211.72]	204.44 [198.95;209.93]	209.43 [207.66;211.20]

© 2024 Stickel AM et al. *JAMA Network Open.*

**Triglycerides** mg/dL	147.40 [143.72;151.08]	159.88 [149.36;170.40]	150.98 [146.40;155.56]
**Glucose, fasting** mg/dL	107.96 [106.52;109.40]	117.13 [113.36;120.91]	109.42 [107.96;110.88]

Note 1: Sample size is unweighted, and all other reported values are weighted to represent the target Hispanic/Latino population

Note 2: Variables are measured at Visit 1

Abbreviations: CESD = Center for Epidemiology Studies Depression Scale; CVD = cardiovascular disease

© 2024 Stickel AM et al. *JAMA Network Open.*

Sensitivity Analyses 1 – Examining associations with hearing operationalized continuously

eTable 2. Associations between hearing sensitivity (pure tone average) at Visit 1 with cognitive performance at Visit 2, on average 7-years later, and cognitive change

Cognitive performance at Visit 2

**Cognitive Outcomes**	**B-SEVLT-Sum**	**B-SEVLT-Recall**	**Word Fluency**	**Digit and Symbol Substitution**	**Trails A**	**Trails B**	**Global Cognition**	**B-SEVLT-Recall / Max of 3 trials**
	**β[CI 95]**	**β[CI 95]**	**β[CI 95]**	**β[CI 95]**	**β[CI 95]**	**β[CI 95]**	**β[CI 95]**	**β[CI 95]**
**Hearing sensitivity**	-0.006	-0.005	-0.005	-0.004	0.005	0.003	-0.005	-0.001
	[-0.009;-0.003]	[-0.008;-0.002]	[-0.008;-0.002]	[-0.007;-0.001]	[0.002;0.008]	[-0.000;0.007]	[-0.007;-0.003]	[-0.004;0.002]
Unweighted N	6091	6080	6072	6031	6036	5816	6091	6086

**Cognitive Outcomes**	**B-SEVLT-Sum**	**B-SEVLT-Recall**	**Word Fluency**	**Digit and Symbol Substitution**	**Trails A**	**Trails B**	**Global Cognition**	**B-SEVLT-Reca Max of 3 trial**
	**β[CI 95]**	**β[CI 95]**	**β[CI 95]**	**β[CI 95]**	**β[CI 95]**	**β[CI 95]**	**β[CI 95]**	**β[CI 95]**
**Hearing sensitivity**	-0.006	-0.005	-0.005	-0.004	0.005	0.003	-0.005	-0.001
	[-0.009;-0.003]	[-0.008;-0.002]	[-0.008;-0.002]	[-0.007;-0.001]	[0.002;0.008]	[-0.000;0.007]	[-0.007;-0.003]	[-0.004;0.002
Unweighted N	6091	6080	6072	6031	6036	5816	6091	6086

Cognitive change from Visit 1 to Visit 2

**Cognitive Outcomes**	**B-SEVLT-Sum**	**B-SEVLT-Recall**	**Word Fluency**	**Digit and Symbol Substitution**	**Global Cognition**	**B-SEVLT-Recall / Max of 3 trials**
	**β[CI 95]**	**β[CI 95]**	**β[CI 95]**	**β[CI 95]**	**β[CI 95]**	**β[CI 95]**
**Hearing sensitivity**	-0.004	-0.004	-0.001	-0.004	-0.003	-0.003
	[-0.008;-0.000]	[-0.008; 0.000]	[-0.004; 0.003]	[-0.008;-0.000]	[-0.007; 0.001]	[-0.007;0.001]
Unweighted N	6061	6054	5976	5910	6078	6053

Note 1: Models adjust for Sex, Age, Education, Heritage, Center, Income, Marital Status, Depressive Symptoms, Framingham Cardiovascular Disease -10yr Risk, Total Cholesterol, Triglycerides, and Fasting Glucose
Note 2: Cognitive outcomes are z-scored
Abbreviations: β = beta estimate; B-SEVLT = Brief- Spanish English Verbal Learning Test; CI =confidence interval; M# = model #; ref = reference

© 2024 Stickel AM et al. *JAMA Network Open.*

eTable 3. Associations between hearing sensitivity (pure tone average) at Visit 1 with mild cognitive impairment prevalence at Visit 2

	**MCI**
	**OR[CI 95]**
**Hearing sensitivity**	1.011
	[1.00;1.02]
Unweighted N	5942

Note 1: Models adjust for Sex, Age, Education, Heritage, Center, Income, Marital Status, Depressive Symptoms, Framingham Cardiovascular Disease -10yr Risk, Total Cholesterol, Triglycerides, and Fasting Glucose
Note 2: Estimates are odds ratios
Abbreviations: MCI = Mild cognitive impairment prevalence; CI =confidence interval; M# = model #; OR = odds ratio; ref = reference

© 2024 Stickel AM et al. *JAMA Network Open.*

Sensitivity Analyses 2 – Repeating analyses to examine robustness to exclusion of individuals who report "ever using" a hearing aid.

eTable 4. Descriptive statistics for the Study of Latinos– Investigation of Neurocognitive Aging excluding hearing aid subsample

	Hearing loss		
	**No (N=5,256)**	**Yes (N=775)**	**Total (N=6,031)**
**Categorical Variables**	**% [CI 95]**	**% [CI 95]**	**% [CI 95]**
**Sex**			
Female	57.12 [55.30;58.94]	39.56 [34.77;44.35]	54.36 [52.65;56.08]
Male	42.88 [41.06;44.70]	60.44 [55.65;65.23]	45.64 [43.92;47.35]
**Education**			
Less than high school	36.74 [34.48;39.00]	46.26 [41.52;51.01]	38.23 [36.13;40.33]
High school or equivalent	21.54 [19.96;23.12]	18.56 [14.36;22.77]	21.07 [19.55;22.59]
More than high school	41.72 [39.50;43.94]	35.17 [29.98;40.37]	40.69 [38.67;42.72]
**Income**			
≤$20000	45.20 [42.73;47.67]	55.86 [50.03;61.70]	46.87 [44.28;49.46]
$20001-$50000	35.36 [33.40;37.33]	29.46 [24.41;34.51]	34.44 [32.49;36.39]
≥$50001	10.88 [9.06;12.69]	4.09 [2.54;5.63]	9.81 [8.21;11.41]
Not Reported	8.56 [7.19;9.93]	10.59 [7.86;13.31]	8.88 [7.62;10.14]
**Marital Status**			
Single	16.96 [15.37;18.54]	15.74 [11.90;19.57]	16.77 [15.28;18.25]
Married/ Living with partner	54.71 [52.16;57.26]	53.96 [48.81;59.10]	54.59 [52.17;57.01]
Separated/ Divorced/ Widowed	28.33 [26.23;30.43]	30.31 [25.95;34.67]	28.64 [26.68;30.60]
**Field Center**			
Bronx	26.87 [23.91;29.83]	23.64 [18.39;28.90]	26.36 [23.40;29.32]
Chicago	12.71 [10.99;14.43]	11.70 [8.81;14.59]	12.55 [10.84;14.26]
Miami	35.33 [30.87;39.79]	43.40 [35.85;50.95]	36.60 [31.98;41.21]
San Diego	25.09 [21.73;28.45]	21.26 [16.59;25.94]	24.49 [21.20;27.78]
**Heritage**			
Dominican	9.51 [7.95;11.07]	6.89 [4.74;9.03]	9.10 [7.61;10.58]
Central American	7.35 [6.10;8.60]	7.70 [5.24;10.17]	7.40 [6.26;8.55]
Cuban	24.88 [21.16;28.60]	31.92 [25.22;38.62]	25.98 [22.18;29.79]
Mexican	34.76 [31.38;38.15]	27.08 [21.72;32.44]	33.56 [30.20;36.92]
Puerto-Rican	14.42 [12.77;16.08]	18.62 [15.05;22.20]	15.08 [13.44;16.72]
South American	5.33 [4.52;6.14]	4.74 [2.64;6.84]	5.24 [4.46;6.02]
Other	3.74 [2.77;4.71]	3.05 [1.18;4.92]	3.64 [2.79;4.48]
**Continuous Variables**	**Mean [CI 95]**	**Mean [CI 95]**	**Mean [CI 95]**
**Age in years**	55.33 [54.96;55.71]	61.37 [60.46;62.28]	56.27 [55.90;56.65]

© 2024 Stickel AM et al. *JAMA Network Open.*

**Depressive symptoms from CESD**	7.31 [7.07;7.56]	7.58 [6.88;8.28]	7.38 [7.14;7.61]
**Framingham CVD - 10yr risk**	14.36 [13.76;14.96]	24.31 [22.34;26.28]	15.96 [15.25;16.68]
**Total cholesterol** mg/dL	209.95 [208.23;211.67]	204.73 [198.82;210.64]	209.45 [207.66;211.23]
**Triglycerides** mg/dL	148.01 [144.28;151.73]	159.91 [149.09;170.73]	151.41 [146.77;156.05]
**Glucose, fasting** mg/dL	108.29 [106.83;109.76]	117.65 [113.37;121.93]	109.74 [108.22;111.25]

Note 1: Sample size is unweighted, and all other reported values are weighted to represent the target Hispanic/Latino population
Note 2: Variables are measured at Visit 1
Abbreviations: CESD = Center for Epidemiology Studies Depression Scale; CVD = cardiovascular disease

© 2024 Stickel AM et al. *JAMA Network Open.*

eTable 5. Associations between hearing impairment at Visit 1 with cognitive performance at Visit 2, on average 7-years later, and cognitive change after exclusion of hearing aid subsample

Cognitive performance at Visit 2

**Cognitive Outcomes**	**B-SEVLT-Sum**	**B-SEVLT-Recall**	**Word Fluency**	**Digit and Symbol Substitution**	**Trails A**	**Trails B**	**Global Cognition**	**B-SEVLT-Recall / Max of 3 trials**
**Hearing impairment**	**β[CI 95]**	**β[CI 95]**	**β[CI 95]**	**β[CI 95]**	**β[CI 95]**	**β[CI 95]**	**β[CI 95]**	**β[CI 95]**
No	ref	ref	ref	ref	ref	ref	ref	ref
Yes	-0.15	-0.12	-0.12	-0.07	0.07	0.03	-0.12	0.03
	[-0.26;-0.05]	[-0.22;-0.02]	[-0.23;-0.00]	[-0.16;0.02]	[-0.04;0.17]	[-0.07;0.13]	[-0.19;-0.05]	[-0.06;0.12]
Unweighted N	6009	5998	5991	5951	5956	5744	6009	6005

Cognitive change from Visit 1 to Visit 2

**Cognitive Outcomes**	**B-SEVLT-Sum**	**B-SEVLT-Recall**	**Word Fluency**	**Digit and Symbol Substitution**	**Global Cognition**	**B-SEVLT-Recall / Max of 3 trials**
	**β[CI 95]**	**β[CI 95]**	**β[CI 95]**	**β[CI 95]**	**β[CI 95]**	**β[CI 95]**
No	ref	ref	ref	ref	ref	ref
Yes	-0.12	-0.1	-0.07	-0.11	-0.12	-0.09
	[-0.25;0.00]	[-0.21;0.00]	[-0.19;0.06]	[-0.22;0.01]	[-0.23;-0.00]	[-0.20;0.03]
Unweighted N	5980	5973	5897	5832	5996	5972

Note 1: Models adjust for Sex, Age, Education, Heritage, Center, Income, Marital Status, Depressive Symptoms, Framingham Cardiovascular Disease -10yr Risk, Total Cholesterol, Triglycerides, and Fasting Glucose
Note 2: Cognitive outcomes are z-scored
Abbreviations: β = beta estimate; B-SEVLT = Brief- Spanish English Verbal Learning Test; CI =confidence interval; M# = model #; ref = reference

© 2024 Stickel AM et al. *JAMA Network Open.*

eTable 6. Associations between hearing impairment at Visit 1 with mild cognitive impairment prevalence at Visit 2 after exclusion of hearing aid subsample

	**MCI**
	**OR[CI 95]**
No	ref
Yes	0.85
	[0.60;1.21]
Unweighted N	5864

Note 1: Models adjust for Sex, Age, Education, Heritage, Center, Income, Marital Status, Depressive Symptoms, Framingham Cardiovascular Disease -10yr Risk, Total Cholesterol, Triglycerides, and Fasting Glucose
Note 2: Estimates are odds ratios
Abbreviations: MCI = Mild cognitive impairment prevalence; CI =confidence interval; M# = model #; OR = odds ratio; ref = reference

© 2024 Stickel AM et al. *JAMA Network Open.*

eTable 7. Associations between hearing operationalized continuously and cognitive performance at INCA and cognitive change, after exclusion of hearing aid subsample

Cognitive performance at Visit 2

Cognitive Outcomes	B-SEVLT-Sum	B-SEVLT-Recall	Word Fluency	Digit and Symbol Substitution	Trails A	Trails B	Global Cognition	B-SEVLT-Recall / Max of 3 trials
	β[CI 95]	β[CI 95]	β[CI 95]	β[CI 95]	β[CI 95]	β[CI 95]	β[CI 95]	β[CI 95]
**Hearing sensitivity**	-0.007	-0.005	-0.005	-0.005	0.005	0.004	-0.006	-0.000
	[-0.01;-0.00]	[-0.01;-0.00]	[-0.01;-0.00]	[-0.01;-0.00]	[0.001;0.009]	[0.001;0.008]	[-0.01;-0.00]	[-0.004;0.003]
Unweighted N	6009	5998	5991	5951	5956	5744	6009	6005

Cognitive change from Visit 1 to Visit 2

Cognitive Outcomes	B-SEVLT-Sum	B-SEVLT-Recall	Word Fluency	Digit and Symbol Substitution	Global Cognition	B-SEVLT-Recall / Max of 3 trials
	β[CI 95]	β[CI 95]	β[CI 95]	β[CI 95]	β[CI 95]	β[CI 95]
**Hearing sensitivity**	-0.005	-0.004	-0.001	-0.004	-0.004	-0.002
	[-0.009;-0.001]	[-0.008;-0.000]	[-0.005;0.003]	[-0.007;0.000]	[-0.008;-0.000]	[-0.007;0.002]
Unweighted N	5980	5973	5897	5832	5996	5972

Note 1: Models adjust for Sex, Age, Education, Heritage, Center, Income, Marital Status, Depressive Symptoms, Framingham Cardiovascular Disease -10yr Risk, Total Cholesterol, Triglycerides, and Fasting Glucose
Note 2: Cognitive outcomes are z-scored
Abbreviations: β = beta estimate; B-SEVLT = Brief- Spanish English Verbal Learning Test; CI =confidence interval; M# = model #; ref = reference

© 2024 Stickel AM et al. *JAMA Network Open.*

eTable 8. Associations between hearing operationalized continuously and MCI, after exclusion of hearing aid subsample

	MCI<br>OR[CI 95]
**Hearing sensitivity**	1.01<br>[1.00;1.02]
Unweighted N	5864

Note 1: Models adjust for Sex, Age, Education, Heritage, Center, Income, Marital Status, Depressive Symptoms, Framingham Cardiovascular Disease -10yr Risk, Total Cholesterol, Triglycerides, and Fasting Glucose
Note 2: Estimates are odds ratios
Abbreviations: MCI = Mild cognitive impairment prevalence; CI =confidence interval; M# = model #; OR = odds ratio; ref = reference

© 2024 Stickel AM et al. *JAMA Network Open.*

Sensitivity Analyses 3 – Examining associations with hearing operationalized using clinical hearing diagnostic criteria

eTable 9. Associations between 3-groups hearing impairment at Visit 1 with cognitive performance at Visit 2, on average 7-years later, and cognitive change

Cognitive performance at Visit 2

**Cognitive Outcomes**	**B-SEVLT Sum**	**B-SEVLT Recall**	**Word Fluency**	**Digit and Symbol Substitution**	**Trails A**	**Trails B**	**Global Cognition**	**B-SEVLT Recall / Max of 3 trials**
**Hearing impairment**	**β[CI 95]**	**β[CI 95]**	**β[CI 95]**	**β[CI 95]**	**β[CI 95]**	**β[CI 95]**	**β[CI 95]**	**β[CI 95]**
Normal	ref	ref	ref	ref	ref	ref	ref	ref
Mild	-0.09	-0.09	-0.02	-0.02	0.05	0.04	-0.06	-0.04
	[-0.18;-0.00]	[-0.17;-0.00]	[-0.10;0.07]	[-0.09;0.05]	[-0.04;0.15]	[-0.04;0.12]	[-0.12;0.01]	[-0.12;0.04]
Moderate+	-0.14	-0.12	-0.18	-0.13	0.34	-0.01	-0.13	-0.02
	[-0.34;0.06]	[-0.29;0.05]	[-0.38;0.02]	[-0.27;0.02]	[0.09;0.59]	[-0.20;0.18]	[-0.27;0.00]	[-0.18;0.13]
Unweighted N	6091	6080	6072	6031	6036	5816	6091	6086

Cognitive change from Visit 1 to Visit 2

**Cognitive Outcomes**	**B-SEVLT Sum**	**B-SEVLT Recall**	**Word Fluency**	**Digit and Symbol Substitution**	**Global Cognition**	**B-SEVLT Recall / Max of 3 trials**
**Hearing impairment**	**β[CI 95]**	**β[CI 95]**	**β[CI 95]**	**β[CI 95]**	**β[CI 95]**	**β[CI 95]**
Normal	ref	ref	ref	ref	ref	ref
Mild	-0.07	-0.06	0.03	-0.04	-0.03	-0.05
	[-0.17;0.03]	[-0.16;0.03]	[-0.07;0.12]	[-0.13;0.05]	[-0.13;0.06]	[-0.15;0.05]
Moderate+	-0.06	-0.08	0.09	-0.10	-0.03	-0.07
	[-0.28;0.15]	[-0.29;0.13]	[-0.12;0.30]	[-0.33;0.12]	[-0.26;0.20]	[-0.30;0.17]
Unweighted N	6061	6054	5976	5910	6078	6053

Note 1: Models adjust for Sex, Age, Education, Heritage, Center, Income, Marital Status, Depressive Symptoms, Framingham Cardiovascular Disease -10yr Risk, Total Cholesterol, Triglycerides, and Fasting Glucose
Note 2: Cognitive outcomes are z-scored
Note 3: Hearing impairment is defined as: Normal (PTA ≤ 20 dB), Mild (20 < PTA ≤40), and Moderate+(>40).
Abbreviations: β = beta estimate; B-SEVLT = Brief- Spanish English Verbal Learning Test; CI =confidence interval; M# = model #; ref = reference

© 2024 Stickel AM et al. *JAMA Network Open.*

eTable 10. Associations between 3-groups hearing impairment at Visit 1 with mild cognitive impairment prevalence at Visit 2

	**MCI**
**Hearing impairment**	**OR[CI 95]**
Normal	ref
Mild	0.90
	[0.66;1.23]
Moderate+	1.39
	[0.77;2.53]
Unweighted N	5942

Note 1: Models adjust for Sex, Age, Education, Heritage, Center, Income, Marital Status, Depressive Symptoms, Framingham Cardiovascular Disease -10yr Risk, Total Cholesterol, Triglycerides, and Fasting Glucose
Note 2: Estimates are odds ratios
Note 3: Hearing impairment is defined as: Normal (PTA ≤ 20 dB), Mild (20 < PTA ≤40), and Moderate+(>40).
Abbreviations: MCI = Mild cognitive impairment prevalence; CI =confidence interval; M# = model #; OR = odds ratio; ref = reference

© 2024 Stickel AM et al. *JAMA Network Open.*

Sensitivity Analyses 4 – Examining associations with hearing operationalized using an alternative definition to reconcile the clinical hearing diagnostic criteria and our original definition for hearing loss

eTable 11. Associations between 3-group hearing impairment at Visit 1 with cognitive performance at Visit 2, on average 7-years later, and cognitive change

Cognitive performance at Visit 2

**Cognitive Outcomes**	**B-SEVLT Sum**	**B-SEVLT Recall**	**Word Fluency**	**Digit and Symbol Substitution**	**Trails A**	**Trails B**	**Global Cognition**	**B-SEVLT Recall / Max of 3 trials**
**Hearing impairment**	**β[CI 95]**	**β[CI 95]**	**β[CI 95]**	**β[CI 95]**	**β[CI 95]**	**β[CI 95]**	**β[CI 95]**	**β[CI 95]**
<=20	ref	ref	ref	ref	ref	ref	ref	ref
>20 to <=25	-0.03	-0.04	0.05	0.00	0.07	0.04	-0.00	-0.08
	[-0.13;0.08]	[-0.14;0.06]	[-0.06;0.16]	[-0.08;0.09]	[-0.06;0.20]	[-0.06;0.13]	[-0.08;0.07]	[-0.20;0.03]
>25	-0.15	-0.13	-0.11	-0.07	0.09	0.03	-0.11	0.00
	[-0.25;-0.05]	[-0.24;-0.03]	[-0.21;0.00]	[-0.15;0.02]	[-0.01;0.19]	[-0.07;0.13]	[-0.19;-0.04]	[-0.09;0.09]
Unweighted N	6091	6080	6072	6031	6036	5816	6091	6086

Cognitive change from Visit 1 to Visit 2

**Cognitive Outcomes**	**B-SEVLT-Sum**	**B-SEVLT-Recall**	**Word Fluency**	**Digit and Symbol Substitution**	**Global Cognition**	**B-SEVLT-Recall / Max of 3 trials**
**Hearing impairment**	**β[CI 95]**	**β[CI 95]**	**β[CI 95]**	**β[CI 95]**	**β[CI 95]**	**β[CI 95]**
<=20	ref	ref	ref	ref	ref	ref
>20 to <=25	-0.02	-0.02	0.11	0.03	0.04	-0.00
	[-0.14;0.10]	[-0.14;0.10]	[-0.01;0.23]	[-0.08;0.14]	[-0.08;0.16]	[-0.12;0.12]
>25	-0.11	-0.11	-0.03	-0.11	-0.10	-0.10
	[-0.23;0.01]	[-0.22;0.01]	[-0.15;0.08]	[-0.22;0.00]	[-0.21;0.02]	[-0.21;0.02]
Unweighted N	6061	6054	5976	5910	6078	6053

Note 1: Models adjust for Sex, Age, Education, Heritage, Center, Income, Marital Status, Depressive Symptoms, Framingham Cardiovascular Disease -10yr Risk, Total Cholesterol, Triglycerides, and Fasting Glucose
Note 2: Cognitive outcomes are z-scored
Abbreviations: β = beta estimate; B-SEVLT = Brief- Spanish English Verbal Learning Test; CI =confidence interval; M# = model #; ref = reference

© 2024 Stickel AM et al. *JAMA Network Open.*

eTable 12. Associations between 3-group hearing impairment at Visit 1 with mild cognitive impairment prevalence at Visit 2

	MCI
Hearing impairment	OR[CI 95]
<=20	ref
>20 to <=25	0.99
	[0.68;1.45]
>25	0.90
	[0.64;1.28]
Unweighted N	5942

Note 1: Models adjust for Sex, Age, Education, Heritage, Center, Income, Marital Status, Depressive Symptoms, Framingham Cardiovascular Disease -10yr Risk, Total Cholesterol, Triglycerides, and Fasting Glucose
Note 2: Estimates are odds ratios
Abbreviations: MCI = Mild cognitive impairment prevalence; CI =confidence interval; M# = model #; OR = odds ratio; ref = reference

© 2024 Stickel AM et al. *JAMA Network Open.*

SUPPLEMENTAL FIGURES

eFigure 1. Analytic sample diagram

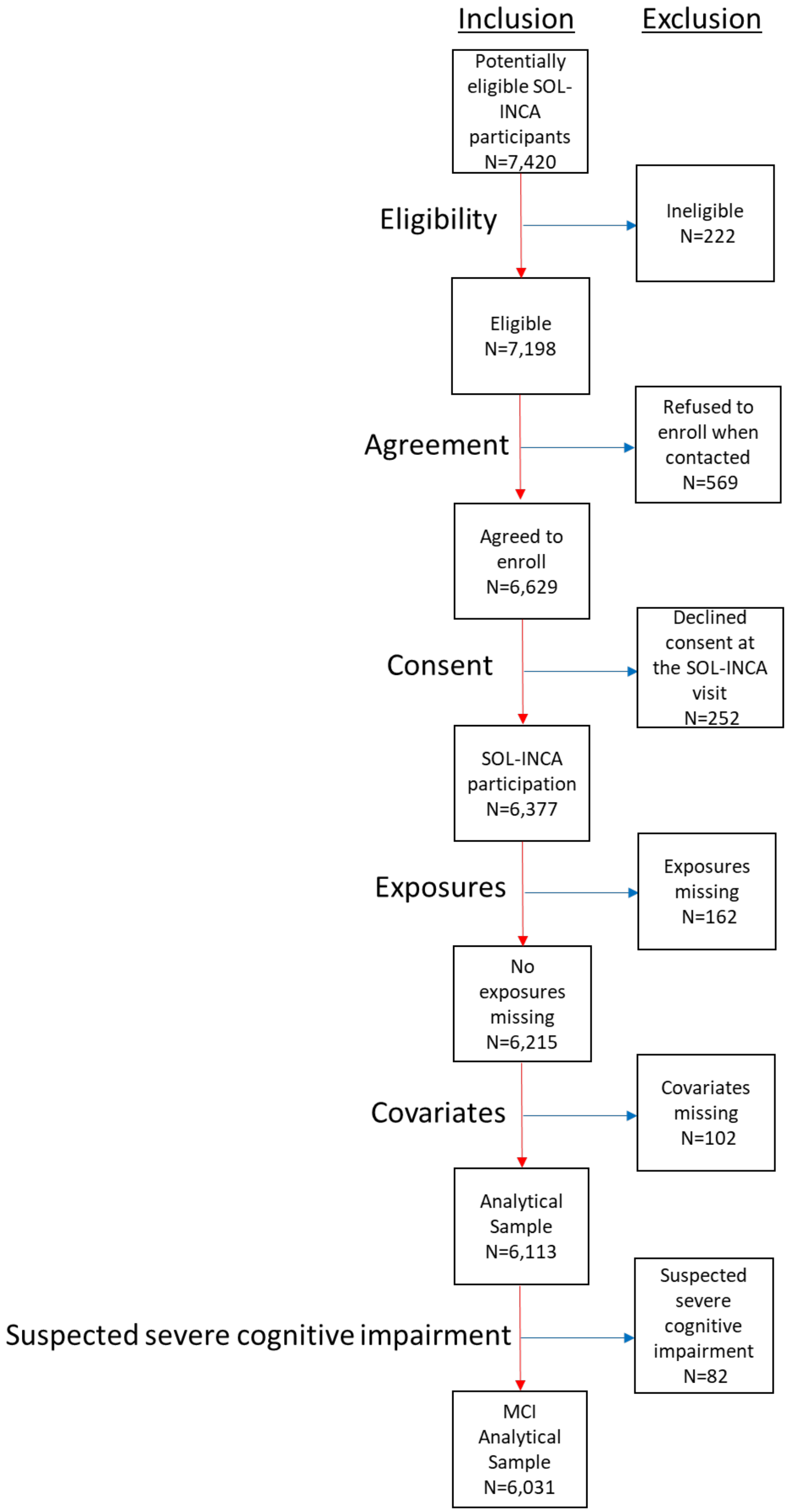

Abbreviations: MCI = mild cognitive impairment; SOL-INCA = Study of Latinos – Investigation of Neurocognitive Aging

© 2024 Stickel AM et al. *JAMA Network Open.*

eFigure 2. Box plots of cognitive performance scores at Visit 2 in the primary analysis

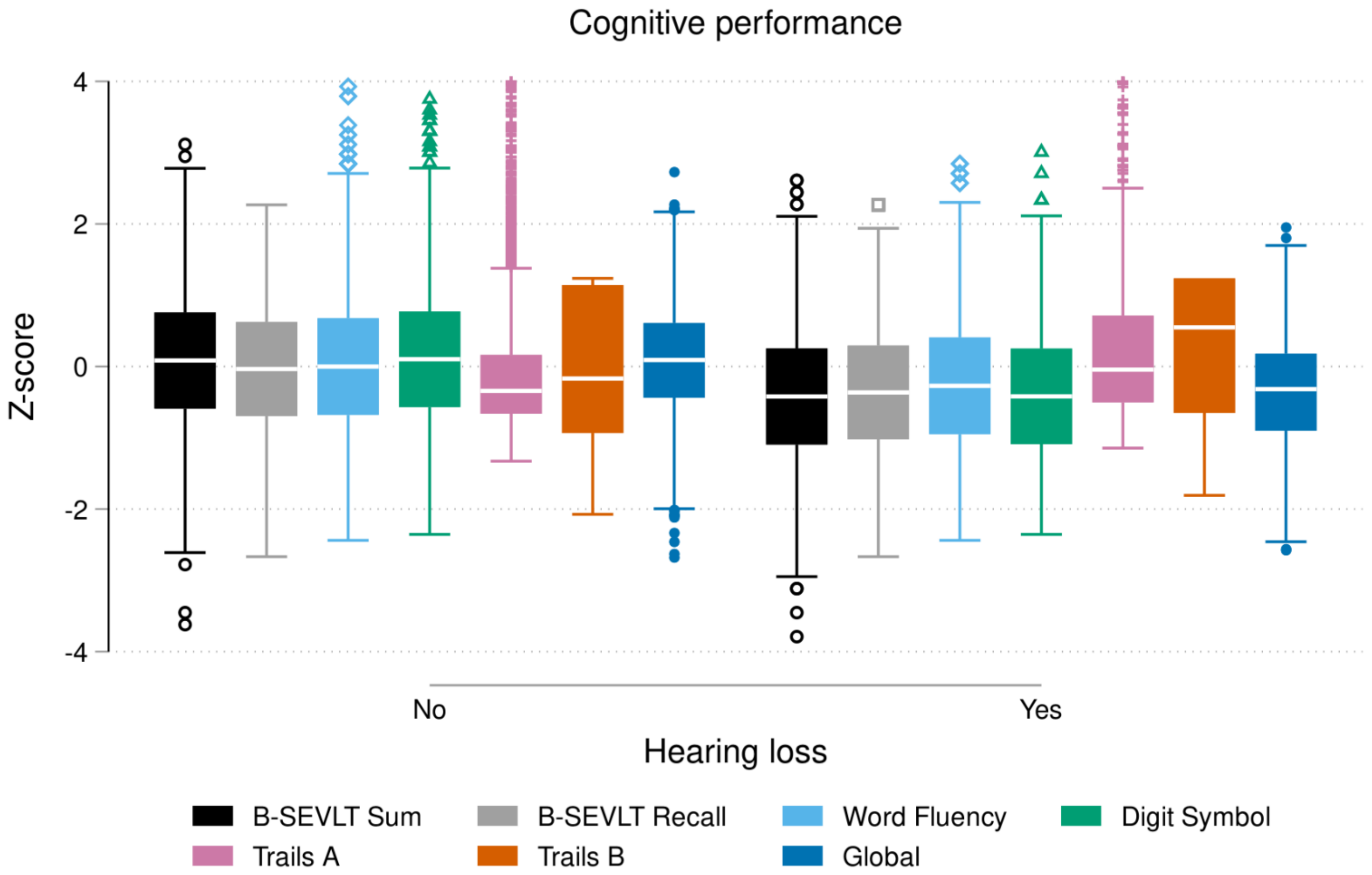

Abbreviations: B-SEVLT = Brief- Spanish English Verbal Learning Test

© 2024 Stickel AM et al. *JAMA Network Open.*

eFigure 3. Box plots of cognitive change scores in the primary analysis

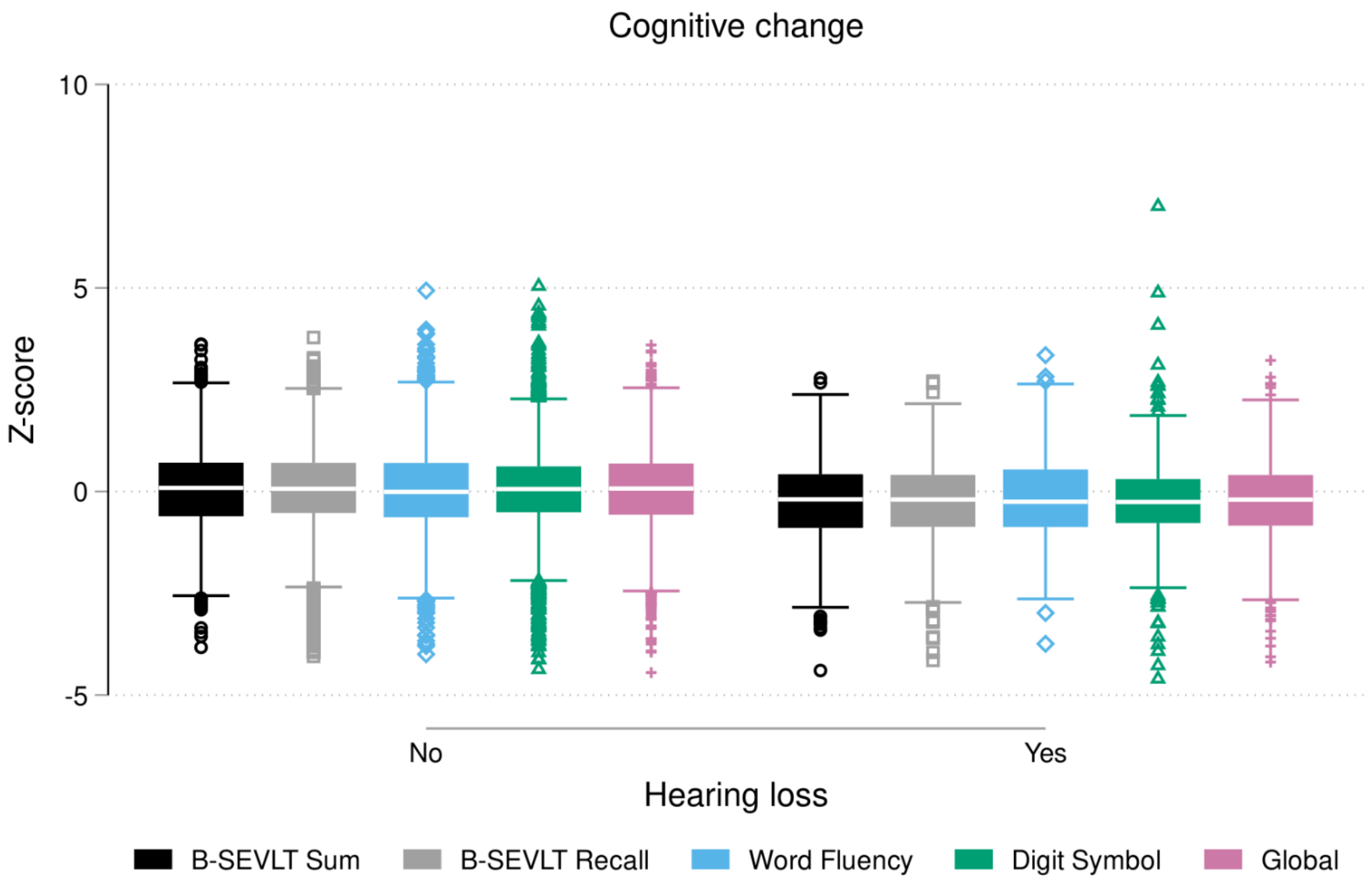

Abbreviation: B-SEVLT = Brief- Spanish English Verbal Learning Test

© 2024 Stickel AM et al. *JAMA Network Open.*
