# Supplementary material for: Hearing Loss and Associated 7-Year Cognitive Outcomes Among Hispanic and Latino Adults
Source: JAMA Otolaryngol Head Neck Surg. 2024 Mar 21;150(5):385–92. doi: 10.1001/jamaoto.2024.0184 (PMC10958383; doi:10.1001/jamaoto.2024.0184)
Supplement: Supplement 2. — Data Sharing Statement [file jamaotolaryngolheadnecksurg-e240184-s002.pdf]

Data Sharing Statement

Data
Data available: No

Additional Information
Explanation for why data not available: Data from the Hispanic Community Health Study/Study of Latinos and its ancillary studies (e.g., the Study of Latinos - Investigation of Neurocognitive Aging) are publicly available through the National Institutes of Health Biologic Specimen and Data Repository Information Coordinating Center (https://biolincc.nhlbi.nih.gov/studies/hchssol/)
